# A Novel Microfluidic Flow Rate Detection Method Based on Surface Plasmon Resonance Temperature Imaging

**DOI:** 10.3390/s16070964

**Published:** 2016-06-24

**Authors:** Shijie Deng, Peng Wang, Shengnan Liu, Tianze Zhao, Shanzhi Xu, Mingjiang Guo, Xinglong Yu

**Affiliations:** State Key Laboratory of Precision Measurement Technology and Instruments, Tsinghua University, Beijing 100084, China; dsj11@mails.tsinghua.edu.cn (S.D.); shengnan_liu@yeah.net (S.L.); ztz12@mails.tsinghua.edu.cn (T.Z.); 13681056064@163.com (S.X.); 13811184011@126.com (M.G.); jyxyxl@mail.tsinghua.edu.cn (X.Y.)

**Keywords:** microfluidic flow rate detection, surface plasmon resonance, temperature variation imaging

## Abstract

A novel microfluidic flow rate detection method based on surface plasmon resonance (SPR) temperature imaging is proposed. The measurement is performed by space-resolved SPR imaging of the flow induced temperature variations. Theoretical simulations and analysis were performed to demonstrate a proof of concept using this approach. Experiments were implemented and results showed that water flow rates within a wide range of tens to hundreds of μL/min could be detected. The flow rate sensor is resistant to disturbances and can be easily integrated into microfluidic lab-on-chip systems.

## 1. Introduction

There is an increasing demand for microfluidic flow rate measurement technologies in various research fields, such as bio-chemical analysis, sample mixing, and environmental monitoring. Various microfluidic flow sensors of different working principles have been proposed in recent years [1,2,3,4,5]; the thermal flow rate sensors working on heat transfer property under the liquid flow, are the most prevalent [6,7]. According to the detection method, thermal flow rate sensors can be classified into three categories: hot element anemometers [8,9], calorimetric flow rate sensors [10,11] and time of flight sensors [12,13]. Calorimetric flow rate sensors which use thermistors that are laid either up- or down-stream of a heater to detect the flow induced temperature distribution, are of the highest sensitivity. The layout of the heater and thermistors is a key design factor that determines the measurement sensitivity and dynamic range [14,15,16]. Once the distance between the heater and thermistors is determined, the measurable flow is limited to a specific dynamic range inversely proportional to sensibility.

To expand the dynamic range and enhance the sensitivity, improvements have been implemented to optimize the arrangement of the heater and thermistors. Several temperature detectors were utilized in the works by N. Sabaté [17] and Seunghyun Kim [18]. The introduction of an array of thermistors facilitated a large expansion of the dynamic range. With the use of fluorescent dyes, Hoera et al. [19] developed a flow sensor based on temperature distribution imaging, that had a wide dynamic range of 10 nL/min to 100 μL/min. Unfortunately, the use of fluorescent dyes impose restrictions on the flow rate sensor in non-labeled detection fields.

Surface plasmon resonance (SPR) is a collective charge density oscillation that occurs at a metal-dielectric interface. As SPR is extremely sensitive to the temperature-dependent refractive index (RI) just above the metal film, it has extensive applications in temperature monitoring and sensing [20,21,22]. In work performed by Chiang et al., a variation as small as 0.027 K was distinguished at 632.8 nm [23]. Furthermore by means of SPR imaging, the refractive index distribution on the whole sensing surface can be simultaneously monitored. When liquid flows through the sensing unit, the temperature above the sensing surface is redistributed. Because the refractive index (RI) is inversely proportional to the temperature, the RI varies along the flow channel, leading to a corresponding variation of the SPR response. Therefore, the liquid flow rate can be resolved from the SPR response variations. 

In this paper, SPR imaging was proposed for the first time to measure the microfluidic flow rate. As the space-resolved SPR imaging is able to monitor the flow-induced continuous temperature variations, rather than several discrete points, this method has more resistance to disturbances and potentially has a wide detection range. As SPR has been widely applied in bio-chemical analysis, the SPR-based flow rate measurement will be facilely integrated into these analytical systems. 

## 2. Theory and Simulations 

### 2.1. Principle

**Figure 1:**
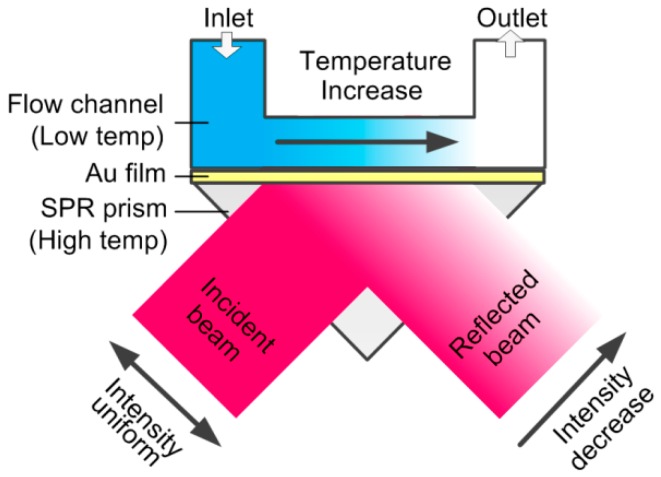
Schematic of the surface plasmon resonance (SPR)-based microfluidic flow rate detection.

The schematic of microfluidic flow rate measurement based on SPR temperature imaging is illustrated in Figure 1. The SPR prism coated with a gold film, together with a flow channel, constitutes the sensing chip. The SPR prism and the water are loaded with high and low temperature, respectively. A collimated beam of light projects onto the bottom of the gold film to initiate SPR. When the water is still, the SPR response (intensity of the reflected beam) is uniform. But when the water is flowing, there will be a temperature gradient along the flow channel. Since the RI is inversely proportional to the temperature, the RI decreases along the flow channel, leading to a corresponding decrease of the SPR response. The decreasing rate varies with the flow rate. As such, the liquid flow rate can be resolved from the variation of the SPR response.

### 2.2. Flow-Induced Temperature Variations

**Figure 2:**
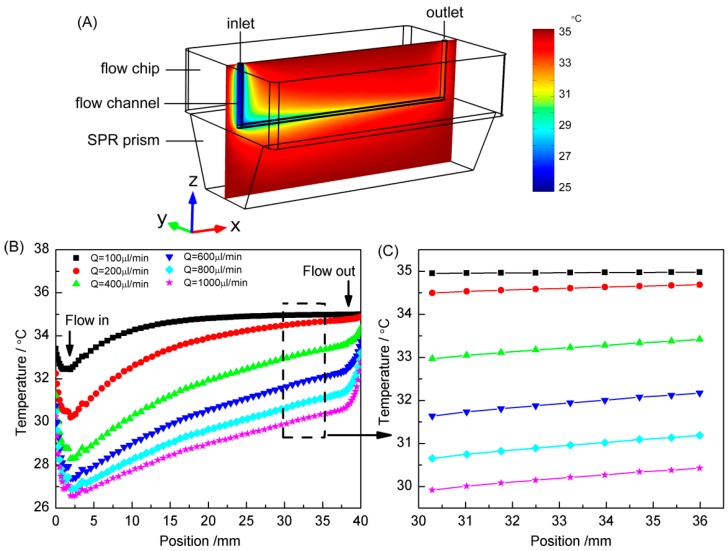
(**A**) Heat map of the SPR sensing chip. (**B**,**C**) the water temperature variations in the flow channel at different flow rates.

The flow-thermal coupling physical field of the sensing chip was simulated using COMSOL. Since SPR is only able to detect the changes above the upper surface of the prism, the flow channel was placed above the prism. For fluid sealing, the flow channel was pressed down by the top Polymethylmethacrylate (PMMA) shell to seal with the bottom prism. Therefore, the flow channel was sandwiched between a top shell and a bottom SPR prism, as illustrated in Figure 2A. In the case where the water and prism were at 25 and 35 °C, respectively, the flow (100 μL/min) induced temperature distribution of the sensing chip is shown in Figure 2A. Due to the temperature difference, heat exchange occurred between the liquid and prism. Figure 2B shows the temperature variations at different flow rates of 100, 200, 400, 600, 800 and 1000 μL/min. Due to the increase of heating time, the liquid temperature increases approximately linearly along streams, except at the inlet and outlet where the flow was unstable. Figure 2C further shows that at slower flow rates, the liquid maintained a higher temperature and a slower rate of temperature increase. Therefore, the section of the flow channel at 30–36 mm (between the two dashed lines) was chosen for flow rate measurement.

### 2.3. Temperature-Induced SPR Response Variations

**Figure 3:**
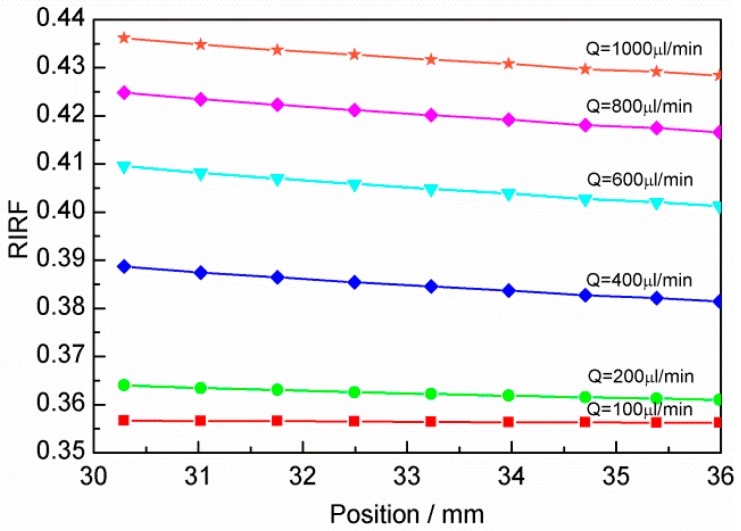
SPR variations in the flow channel at different flow rates.

Due to the dependence of the refractive index on the temperature, the flow-induced temperature gradient generates a variation of the SPR response. Based on the multi-layer model, Fresnel equations [24] were used to analyze the SPR response. Figure 3 depicts the corresponding variations of the refractive index related factor (*RIRF*). The *RIRF* variation shows an inverse relationship with the liquid temperature variation. As the *RIRF* varies approximately linearly and its variation rate Δ*RIRF*/Δ*l* changes at different flow rates, the Δ*RIRF*/Δ*l* can be used to quantify the flow rate.

### 2.4. Effect of the Liquid-Prism Temperature Difference

**Figure 4:**
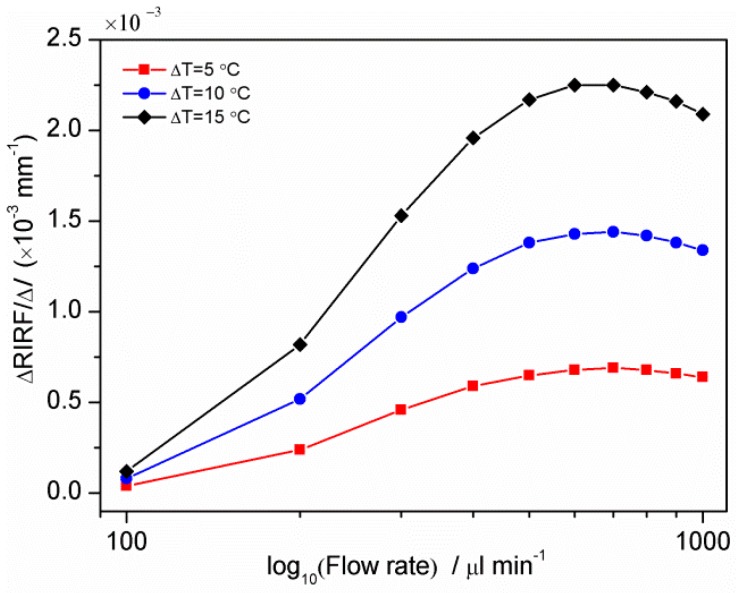
Relationship between the Δ*RIRF*/Δ*l* and flow rate at different water-prism temperature differences.

The temperature difference between the liquid and prism is a key factor affecting the prism-liquid heat exchange, leading to different temperature variation rates. To research the effect of the liquid-prism temperature on the measurement, the water temperature was fixed at 25 °C while the prism temperature was changed to 30, 35 and 40 °C, respectively. The relationship between the Δ*RIRF*/Δ*l* and the logarithm of flow rate is shown in Figure 4. It can be seen that the detection range is about 100–500 μL/min, with the linear range also being approximately 100–500 μL/min. When the fluid is faster than 500 μL/min, the temperature variations in the monitoring section of the flow channel are hardly distinguishable. Therefore the upper limit of the linear range is 500 μL/min. The lower limit of 100 μL/min was chosen due to the performance of the pumping system, and not due to limitations of the sensor. With larger liquid-prism temperature differences, the sensitivity of the measurement was enhanced.

## 3. Experiments

### 3.1. SPR Imaging System and Sensing Chip

**Figure 5:**
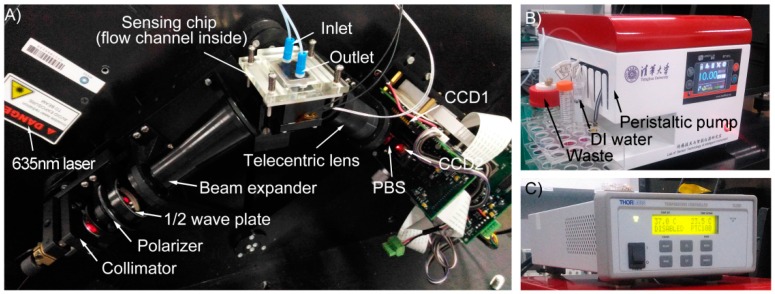
Photographs of the SPR imaging system, consisting of the SPR optic system (**A**), the medium injection system (**B**) and the temperature controller (**C**).

The SPR experiments were performed using a home-built SPR differential interferometric imaging system shown in Figure 5A. This SPR imaging system has been discussed in a previous publication [25]. Briefly, a p-polarized and collimated beam of light was projected onto a Kretschmann prism-based sensing chip. The reflected light was split into two orthogonal-polarized light beams and imaged onto two CCD cameras. Two interferograms of 180° phase-difference were sequentially obtained.

A Poly (dimethylsiloxane) (PDMS) sheet with a channel of 0.5 × 1 mm^2^ cross-section was pressed and sealed with the sensing chip, forming the flow channel. The sensing chip was positioned into an aluminum holder, in which a PT100 resistance thermometer detector (RTD) and a polyimide heating film were embedded. A PID temperature controller (TC200, Thorlabs, Shanghai, China) was used to control the sensing chip temperature.

### 3.2. Experimental Procedures

A self-built injection system was used to generate different flow rates of water, as shown in Figure 5B. The system consisted of a peristaltic pump and a pulsation dampener to acquire a stable flow. The flow rate was corrected by a commercial flow rate sensor (SLI-2000, Sensirion AG, Staefa, Switzerland). Prior to experiments, the prism was preheated overnight to achieve the expected temperature (30, 35, or 40 °C). Then deionized water (DI, ~25 °C) was injected into the flow channel at 50 μL/min. The injection lasted for 20 min to achieve a stable temperature distribution. Then the water flow was stopped for roughly 30 min to recover the initial temperature distribution for the prism. The measurement of the water flow was repeated three times. Water flows of 100, 200, 400, 600, 800, 1000 μL/min was measured using the above procedure. 

### 3.3. Data Processing 

A time series of *RIRF* distribution images of the sensing surface was sequentially extracted from the two interferograms. The temperature distributions in the flow channel were included in the *RIRF* images. To quantify the SPR response variation along the flow streams, the *RIRF* was averaged widthwise and then the average *RIRF* along the flow channel was plotted using a linear fit with the distance from the inlet. The *RIRF* variation rate was used to quantify the flow rates.

## 4. Results and Discussion

### 4.1. SPR Response Distribution across the Sensing Surface

**Figure 6:**
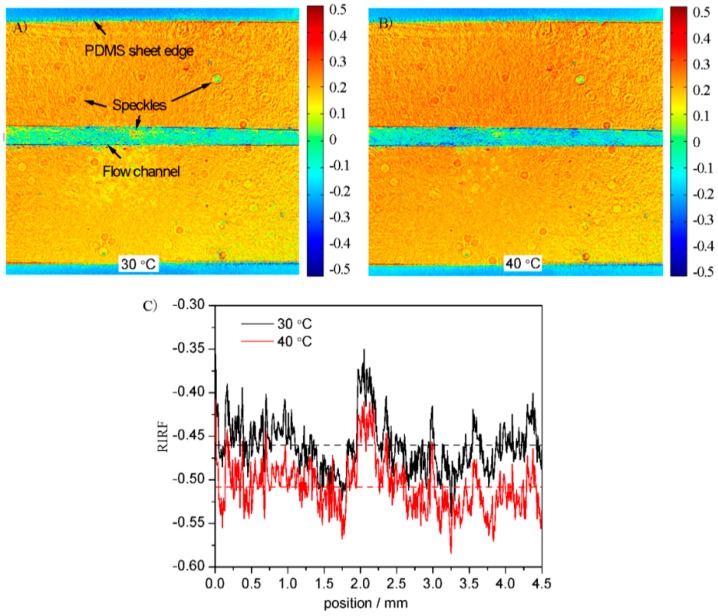
*RIRF* distributions across the sensing surface with the water temperature of 30 °C (**A**) and 40 °C (**B**). The *RIRF* variations in the flow channel (**C**).

It is necessary to test the SPR response distribution across the sensing surface before measuring its variations. Figure 6 shows the *RIRF* distribution across the sensing surface when the water was 30 and 40 °C, respectively. It is obvious that the SPR response was uniform except for some speckles in the flow channel. To quantify the SPR response variation along the flow channel, the response was averaged widthwise and the average response along the flow stream was shown in Figure 6C. It can be seen that the SPR response was relatively unchanged except for the disturbance of some speckles. This verified that SPR imaging was able to measure the flow-induced SPR response variations.

### 4.2. SPR Response Variations with Temperature 

**Figure 7:**
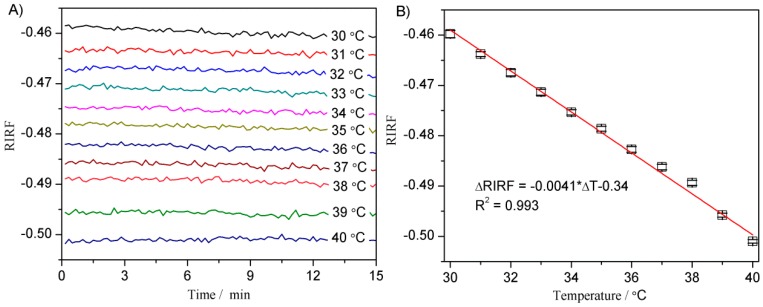
The *RIRF* variations versus time at different temperatures (**A**) and the relationship between *RIRF* and temperature (**B**).

The refractive index (RI) is sensitive to temperature. To verify the influence of temperature on the SPR response, the flow channel was filled with DI water of a temperature varying from 30 to 40 °C and each temperature change lasted 15 min. Figure 7A presents the SPR response of water at different temperatures. It shows that the *RIRF* was inversely proportional to the water temperature, consistent with the works of Moreira [26]. Figure 7B shows the fitted linear relationship between the *RIRF* and the water temperature. It can be fitted linearly to Δ*RIRF* = 0.0041 × Δ*T* + 0.34, with the standard deviation R^2^ = 0.993. 

### 4.3. Flow-Induced SPR Response Variations 

The above mentioned experiments have proven that the SPR response has a linear relationship with the water temperature. The flow-induced temperature variations along the flow channel were then measured. Water was injected into the flow channel using flow rates of 50, 100, 200, 400, 600, 800, 1000 μL/min and the induced temperature distributions were detected sequentially. 

**Figure 8:**
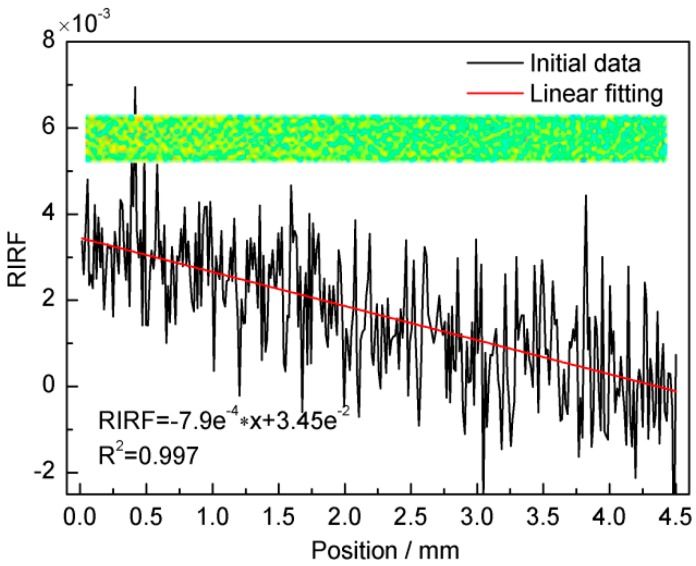
The *RIRF* distribution in the flow channel (inset) and the widthwise-averaged *RIRF* variation along streams at 50 μL/min.

Figure 8 shows the *RIRF* distribution (inset) of a 50 μL/min water flow rate and the subsequent signal processing. It can be seen from the inset that the *RIRF* decreased slightly along the flow stream. This occurred because the water flow was heated by the prism and thus the temperature increased gradually at it moved downstream. Due to the negative linear relationship between the temperature and *RIRF*, the *RIRF* decreased along the streams. The widthwise-averaged *RIRF* variations along the flow streams were fitted linearly with a decreasing rate of nearly −7.9 × 10^−4^ *RIRF*/mm. 

**Figure 9:**
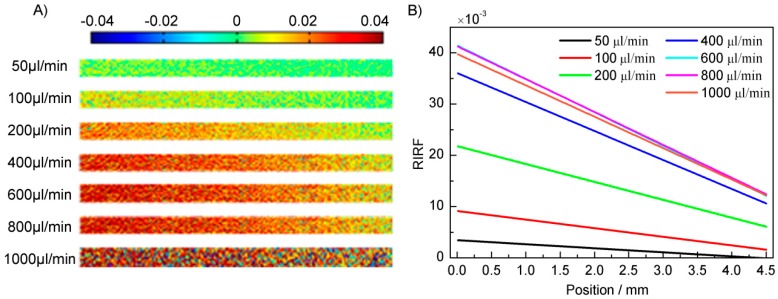
The RIRF distributions (**A**) and the linear fittings of Δ*RIRF*/Δ*l* (**B**) at different flow rates.

The *RIRF* distributions and processed results of other flow rates are presented in Figure 9. It shows that higher flow rates result in larger *RIRF* and larger *RIRF* variation rate. Lower flow rates prolong the heating time of the water flow, resulting in a higher temperature at the monitoring area. As the *RIRF* is negatively proportional to temperature, *RIRF* increased with the flow rate. In addition, it is possible that the temperature of slower water flow has reached approximately the equilibrium temperature, while the water of higher flow was still heated and thus generated high *RIRF* variation rate in the SPR monitoring area. This was consistent with the previous COMSOL simulations.

**Figure 10:**
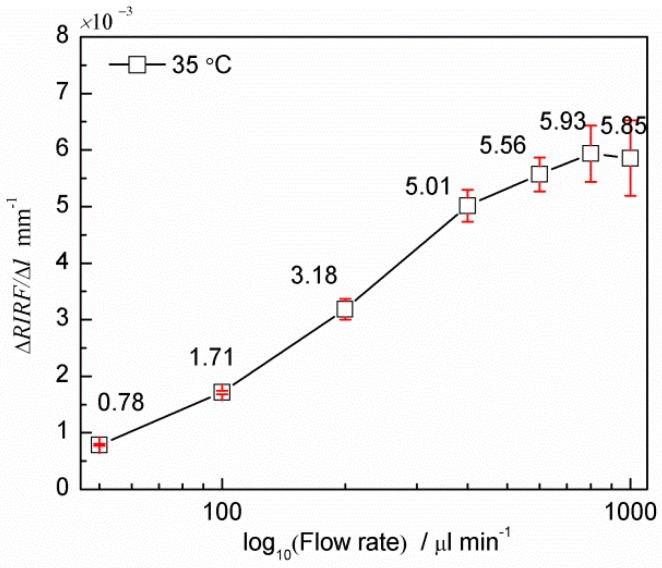
SPR response variation (Δ*RIRF*/Δ*l*) vs. flow rate (Q).

Figure 10 presents the extracted *RIRF* variation rate, Δ*RIRF*/Δ*l*, at different flow rates. It shows that the variation rate increased approximately linearly with the logarithm of flow rate over the range of 50–800 μL/min. Three experiments were performed and the results followed a similar trend with less than 11% relative standard deviation. This demonstrates the proof of concept of flow rate detection based on SPR imaging.

For thermal flow rate sensors, the detection range and sensitivity are mainly determined by the layout of the heating element and the temperature measurement elements. Increasing the distance between the heating and temperature measurement elements expands the detection range, but also reduces the sensitivity. While most existing flow sensors can only measure the temperature of several discrete points, SPR imaging can measure continuous space-resolved temperature variations. Therefore, it is possible for a SPR-based flow sensor to achieve an extremely wide detection range with the same device by selecting different temperature sensing regions. Here it should be mentioned that the lowest limit of 50 μL/min was chosen due to the performance of the pumping system, and not due to limitations of the sensor.

### 4.4. Influence of the Prism-Liquid Temperature Difference 

**Figure 11:**
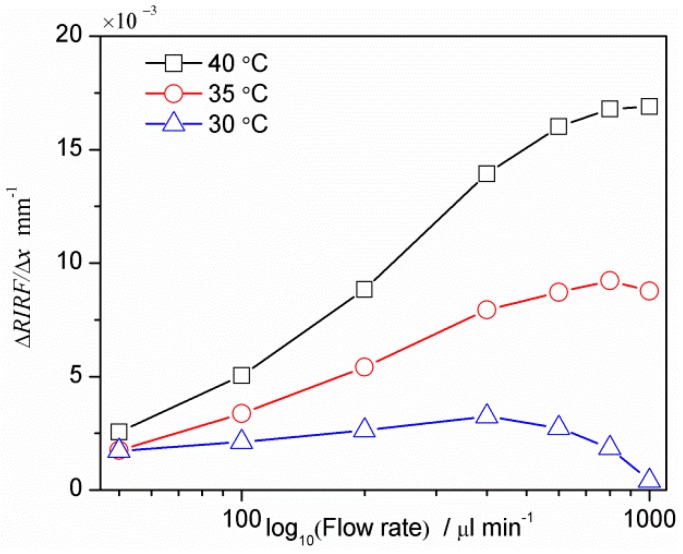
SPR response variation (Δ*RIRF*/Δ*l*) vs. flow rate (Q) with the prism temperature of 30, 35 and 40 °C.

To research the influence of the prism-liquid temperature difference on the measurement, water flow rates with different prism-liquid temperature difference were detected. Figure 11 shows the relationship between the SPR response variation rate Δ*RIRF*/Δ*l* and the logarithm of flow rate, where the water was 25 °C and the prism was 30, 35 and 40 °C, respectively. It can be seen that larger temperature differences lead to higher detection sensitivity, consistent with the previous theoretical analysis. Therefore, a larger temperature difference is desirable to enhance the sensitivity. However in the case of temperature sensitive experiments, such as biological studies, suitable temperature differences are necessary to balance the sensitivity and the object under test.

Compared with the conventional thermal flow sensor, which is of high integration based on the MEMS technology, the SPR-based flow sensor is more complex due to its optical system for space-resolved temperature sensing. In this paper, the SPR system used several optical elements for interferometric imaging to improve its performance, further lowering the integration barrier of the flow sensor. However, complex interferometric imaging is unnecessary for flow rate detection, where SPR intensity imaging which uses less optical elements, is sufficient. Based on the proof of concept of the SPR-based flow sensor that has been demonstrated in this paper, a new compact flow sensor based on SPR intensity imaging has been designed and is under development. This should allow for integration of the SPR-based flow sensor that will be greatly improved. As SPR has been widely applied in bio-chemical analysis, this method should also be easily integrated into these analytical systems.

## 5. Conclusions

In this paper, a microfluidic flow rate detection method based on SPR temperature imaging is presented. The proof of concept has been demonstrated through theoretical simulations and experiments. The novel calorimetric flow rate measurement method allows reliable monitoring of liquid flow rates in a wide range of tens to hundreds of μL/min. As SPR has been commonly used for bio-chemical analysis, this novel SPR-based flow rate detection method should also be easily integrated into these systems. Other than water-prism temperature difference, some factors such as liquids of different heat capacities and the flow channel dimensions need to be further researched. Furthermore, the performance of the flow sensor needs to be enhanced, including improving its integration and expanding the detection range for biological applications.

## Abbreviations

The following abbreviations are used in this manuscript:

SPR	surface plasmon resonance
RI	refractive index
RIRF	refractive index related factor
CCD	charge coupled device
PMMA	Polymethylmethacrylate

## References

[1] Li Y., Yan G., Zhang L., He S. (2015). Microfluidic flowmeter based on micro “hot-wire” sandwiched Fabry-Perot interferometer. Opt. Express.

[2] Vilares R., Hunter C., Ugarte I., Aranburu I., Berganzo J., Elizalde J., Fernandez L.J. (2010). Fabrication and testing of a SU-8 thermal flow sensor. Sens. Actuators B Chem..

[3] Lien V., Vollmer F. (2007). Microfluidic flow rate detection based on integrated optical fiber cantilever. Lab Chip.

[4] Sengupta S., Ziaie B., Barocas V.H. (2004). Lag-after-pulsed-separation microfluidic flowmeter for biomacromolecular solutions. Sens. Actuators B Chem..

[5] Collins J., Lee A.P. (2004). Microfluidic flow transducer based on the measurement of electrical admittance. Lab Chip.

[6] Ahrens R., Schlote-Holubek K. (2009). A micro flow sensor from a polymer for gases and liquids. J. Micromech. Microeng..

[7] Ernst H., Jachimowicz A., Urban G.A. (2002). High resolution flow characterization in Bio-MEMS. Sens. Actuators A Phys..

[8] Caldas P., Jorge P.A., Rego G., Frazão O., Santos J.L., Ferreira L.A., Araújo F. (2011). Fiber optic hot-wire flowmeter based on a metallic coated hybrid long period grating/fiber Bragg grating structure. Appl. Opt..

[9] Van Oudheusden B.W. (1999). The determination of the effective ambient temperature for thermal flow sensors in a non-isothermal environment. Sens. Actuators A Phys..

[10] Dijkstra M., de Boer M.J., Berenschot J.W., Lammerink T., Wiegerink R.J., Elwenspoek M. (2008). Miniaturized thermal flow sensor with planar-integrated sensor structures on semicircular surface channels. Sens. Actuators A Phys..

[11] Kim T.H., Kim S.J. (2006). Development of a micro-thermal flow sensor with thin-film thermocouples. J. Micromech. Microeng..

[12] Berthet H., Jundt J., Durivault J., Mercier B., Angelescu D. (2011). Time-of-flight thermal flowrate sensor for lab-on-chip applications. Lab Chip.

[13] Chung J., Grigoropoulos C.P., Greif R. (2003). Infrared thermal velocimetry for nonintrusive flow measurement in silicon microfluidic devices. Rev. Sci. Instrum..

[14] Kuo J.T., Yu L., Meng E. (2012). Micromachined thermal flow sensors—A review. Micromachines.

[15] Wu S., Lin Q., Yuen Y., Tai Y. (2001). MEMS flow sensors for nano-fluidic applications. Sens. Actuators A Phys..

[16] Yan W., Liu C., Li J., Ma L., Niu D. (2005). Thermal distribution microfluidic sensor based on silicon. Sens. Actuators B Chem..

[17] Sabaté N., Santander J., Fonseca L., Gràcia I., Cané C. (2004). Multi-range silicon micromachined flow sensor. Sens. Actuators A Phys..

[18] Kim S., Nam T., Park S. (2004). Measurement of flow direction and velocity using a micromachined flow sensor. Sens. Actuators A Phys..

[19] Hoera C., Skadell M.M., Pfeiffer S.A., Pahl M., Shu Z., Beckert E., Belder D. (2016). A chip-integrated highly variable thermal flow rate sensor. Sens. Actuators B Chem..

[20] Wagner C.E., Macedo L.J., Opdahl A. (2015). Temperature gradient approach for rapidly assessing sensor binding kinetics and thermodynamics. Anal. Chem..

[21] Srivastava S.K., Gupta B.D. (2010). Simulation of a localized surface-plasmon-resonance-based fiber optic temperature sensor. JOSA A.

[22] Turhan-Sayan G. (2003). Temperature effects on surface plasmon resonance: Design considerations for an optical temperature sensor. J. Lightwave Technol..

[23] Chiang H., Yeh H., Chen C., Wu J., Su S., Chang R., Wu Y., Tsai D.P., Jen S.U., Leung P.T. (2004). Surface plasmon resonance monitoring of temperature via phase measurement. Opt. Commun..

[24] Ekgasit S., Thammacharoen C., Yu F., Knoll W. (2004). Evanescent field in surface plasmon resonance and surface plasmon field-enhanced fluorescence spectroscopies. Anal. Chem..

[25] Wang D., Ding L., Zhang W., Luo Z., Ou H., Zhang E., Yu X. (2012). A high-throughput surface plasmon resonance biosensor based on differential interferometric imaging. Meas. Sci. Technol..

[26] Moreira C.S., Lima A., Neff H., Thirstrup C. (2008). Temperature-dependent sensitivity of surface plasmon resonance sensors at the gold-water interface. Sens. Actuators B Chem..

